# Labor induction *vs* expectant management at 40 weeks: association with Cesarean delivery and intrapartum fetal compromise and role of maternal age and cerebroplacental ratio

**DOI:** 10.1002/uog.70350

**Published:** 2026-10-16

**Authors:** J. Morales‐Roselló, A. Khalil, A. Martínez‐Varea

**Affiliations:** ^1^ Obstetrics and Gynecology Department La Fe University and Polytechnic Hospital Valencia Spain; ^2^ Department of Pediatrics, Obstetrics and Gynecology University of Valencia Valencia Spain; ^3^ Fetal Medicine Unit, St George's University Hospitals NHS Foundation Trust University of London London UK; ^4^ Vascular Biology Research Centre Molecular and Clinical Sciences Research Institute, St George's University of London London UK; ^5^ Fetal Medicine Unit, Liverpool Women's Hospital University of Liverpool Liverpool UK; ^6^ Facultad de Ciencias de la Salud Universidad (CEU) Cardenal Herrera Valencia Spain

**Keywords:** adverse perinatal outcome, cerebroplacental ratio, Cesarean section, induction of labor

## Abstract

**Objectives:**

To evaluate the clinical course of pregnancies reaching the estimated due date and to determine the relative contribution of maternal and fetal factors, particularly labor induction, fetal Doppler status and maternal age, to the risk of adverse outcome and Cesarean delivery (CD).

**Methods:**

This was a retrospective observational study of singleton pregnancies that reached 40 + 0 weeks' gestation at a single center between December 2020 and December 2025. Maternal characteristics, fetal ultrasound and Doppler indices and delivery outcomes were collected. Variables were compared between pregnancies managed with induction of labor and those managed expectantly at 40 + 0 to 40 + 6 weeks, and between pregnancies undergoing induction of labor and those with spontaneous onset of labor at any gestational age ≥ 40 + 0 weeks. Multivariable logistic regression was used to assess the associations of study variables with intrapartum fetal compromise (IFC), CD for abnormal cardiotocography (CD‐CTG) and CD for failure to progress (CD‐FP). Effect sizes were expressed as adjusted odds ratios (aORs) with 95% CI.

**Results:**

Among 739 pregnancies reaching 40 weeks' gestation, 102 (13.8%) underwent induction of labor at 40 + 0 to 40 + 6 weeks, while 637 (86.2%) were initially managed expectantly. Overall, 393 (53.2%) cases had spontaneous onset of labor and 346 (46.8%) were induced at ≥ 40 + 0 weeks. The rate of CD was significantly higher among pregnancies undergoing induction compared with those managed expectantly at 40 + 0 to 40 + 6 weeks (*P* < 0.0001) and with those who progressed to spontaneous onset of labor at any gestational age ≥ 40 + 0 weeks (*P* < 0.0001). On multivariable analysis, induction of labor and cerebroplacental ratio (CPR) multiples of the median (MoM) were independently associated with both IFC (aOR, 2.30 (95% CI, 1.20–4.39); *P* = 0.0116 and 0.07 (95% CI, 0.02–0.24); *P* < 0.0001, respectively) and CD‐CTG (aOR, 3.10 (95% CI, 1.46–6.56); *P* = 0.0032 and 0.05 (95% CI, 0.01–0.20); *P* < 0.0001, respectively). Parity (aOR, 0.48 (95% CI, 0.32–0.72); *P* = 0.0004), estimated fetal weight centile (aOR, 1.01 (95% CI, 1.00–1.02); *P* = 0.0018) and induction of labor (aOR, 2.39 (95% CI, 1.54–3.73); *P* = 0.0001) were associated independently with CD‐FP. Maternal age was not associated independently with CD‐FP, and its borderline associations with IFC and CD‐CTG were no longer significant after adjustment for CPR MoM.

**Conclusions:**

Induction of labor was associated with higher rates of CD at ≥ 40 + 0 weeks, whereas maternal age was not associated independently with adverse intrapartum outcome after adjustment for CPR MoM. Lower CPR MoM was associated independently with IFC and CD‐CTG. These findings support further evaluation of individualized management strategies incorporating fetal Doppler assessment in these pregnancies. © 2026 The Author(s). *Ultrasound in Obstetrics & Gynecology* published by John Wiley & Sons Ltd on behalf of International Society of Ultrasound in Obstetrics and Gynecology.

## INTRODUCTION

Induction of labor at or before the estimated due date has been proposed as a strategy to mitigate adverse perinatal outcome without increasing the risk of Cesarean delivery (CD)^1^, ^2^, ^3^, ^4^. This approach has been adopted increasingly, particularly among pregnant women of advanced maternal age^5^, ^6^. Despite the strength of evidence from randomized trials, uncertainty remains regarding the impact of routine early induction on the natural course of pregnancy, and observational studies have reported conflicting associations between induction of labor and CD across different risk groups, gestational ages (GAs) and maternal ages^7^, ^8^, ^9^, ^10^. Furthermore, fetal Doppler parameters, particularly the cerebroplacental ratio (CPR), have not been included in these analyses, despite their relevance in identifying fetuses at risk of adverse outcome and reduced tolerance to labor in late gestation^11^, ^12^, ^13^, ^14^.

The aim of this study was to evaluate the maternal and fetal determinants of adverse outcome and CD beyond the estimated due date using multivariable analysis, with a focus on labor induction, advanced maternal age and fetal Doppler assessment.

## METHODS

### Study design and population

This was a retrospective observational study of singleton pregnancies managed at the Hospital Universitario y Politécnico La Fe, Valencia, Spain, between December 2020 and December 2025. Eligible cases were pregnancies that reached 40 + 0 weeks' gestation and subsequently went into labor, either spontaneously or after induction. Although all pregnancies were classified as low risk, induction at 40–41 weeks was indicated in some cases, most commonly owing to prelabor rupture of membranes, post‐term pregnancy, abnormal fetal surveillance or advanced maternal age. According to the institutional protocol, pregnancies beyond 40 weeks underwent close surveillance with Doppler ultrasound and cardiotocography (CTG), with a maximum interval of 7 days between examinations. Only the examination closest to delivery was included in the analysis. Exclusion criteria were multiple gestation, stillbirth, elective CD without labor and incomplete data. Ethical approval for this study was obtained from the local institutional review board (Instituto de Investigación Sanitaria La Fe, reference 2014/0063). As this was a retrospective study, informed consent was not required.

### Data collection

Maternal, fetal and perinatal data were obtained from clinical records and the last ultrasound examination before delivery. Maternal and pregnancy characteristics collected included maternal age, parity, pregestational weight and height, GA at ultrasound examination, fetal sex, estimated fetal weight (EFW), fetal middle cerebral artery (MCA) pulsatility index (PI), umbilical artery (UA)‐PI and CPR.

Perinatal outcomes included onset of labor (spontaneous or induced), GA at delivery, days from ultrasound examination to delivery, mode of delivery (spontaneous vaginal, assisted vaginal or CD), birth weight, 5‐min Apgar score, UA pH and intrapartum fetal compromise (IFC).

### Ultrasound and Doppler assessment

EFW was calculated using standard fetal biometry (head circumference, biparietal diameter, abdominal circumference and femur length) according to Hadlock's four‐parameter formula. UA and MCA Doppler were assessed using color and pulsed‐wave Doppler. The MCA was sampled near the circle of Willis at the level of the sphenoid wing, and the UA was sampled in a free‐floating loop of the umbilical cord. The CPR was calculated as MCA‐PI divided by UA‐PI. All measurements were obtained during fetal quiescence, in the absence of tachycardia and with an optimized insonation angle, using Voluson ultrasound systems (GE Healthcare, Zipf, Austria), equipped with 2–8‐MHz convex transducers.

EFW and birth weight were converted to centiles using local population reference charts, adjusted for fetal sex^15^. Small‐for‐gestational age (SGA) was defined as birth weight < 10^th^ centile, and large‐for‐gestational age (LGA) was defined as birth weight > 90^th^ centile. Doppler indices (MCA‐PI, UA‐PI and CPR) were expressed as multiples of the median (MoM) using GA‐specific reference values^16^, ^17^, calculated as follows:

$$\begin{array}{ll} & \mathrm{MCA}‐\mathrm{PI}\;50^{\mathrm{th}}\;\text{centile}=-3.266164164\\ & \;+\left(0.368135209\times \mathrm{GA}\right)-(0.005251488\times {\mathrm{GA}}^{2}) \end{array}$$

$$\begin{array}{ll} & \mathrm{UA}‐\mathrm{PI}\;50^{\mathrm{th}}\;\text{centile}=2.2037-\left(0.057955\times \mathrm{GA}\right)\\ & \;+(0.00053953\times {\mathrm{GA}}^{2}) \end{array}$$

$$\begin{array}{ll} & \text{CPR}\;50^{\mathrm{th}}\;\text{centile}=-3.814786276\\ & \;+\left(0.36363249\times \mathrm{GA}\right)-(0.005646672\times {\mathrm{GA}}^{2}) \end{array}$$

### Labor management

Management followed institutional protocols^18^, ^19^, ^20^. Induction was performed using oxytocin (Syntocyion®; Alfasigma S.p.A., Pomezia (Rome), Italy) when the cervix was favorable. In the case of an unfavorable cervix, cervical ripening was achieved using prostaglandin E2 (dinoprostone, Propess®; Ferring GmbH, Kiel, Germany) or a mechanical balloon catheter (Cook®; Cook Medical LLC, Bloomington, IN, USA) for up to 12 h. Mechanical methods were preferred for women that had undergone a previous CD and those with suspected fetal smallness. If labor did not start, oxytocin was administered subsequently. Neonatal status was assessed using the 5‐min Apgar score and UA pH.

### Definition of outcomes

The primary outcomes were CD for failure to progress (CD‐FP), CD for abnormal cardiotocography (CD‐CTG) and IFC.

CD‐FP was defined according to national and institutional criteria: (a) failed induction: failure to reach active labor (≥ 4 cm dilation with complete effacement) after ≥ 12 h of oxytocin with adequate uterine activity (3–5 contractions per 10 min); (b) arrest of labor: no cervical change for ≥ 4 h during active labor; or (c) cephalopelvic disproportion: arrest of fetal descent for ≥ 2–3 h after full dilation (depending on parity). The thresholds were extended by 1 h in the presence of epidural analgesia. Protracted labor was not considered a failure if vaginal delivery was achieved. CD‐CTG was defined as CD indicated for abnormal CTG^18^ or fetal scalp pH < 7.20^19^. IFC included cases that underwent CD‐CTG^18^, ^19^ or had UA pH < 7.10 at birth. This variable was considered to represent adverse perinatal outcome.

### Statistical analysis

Continuous variables are presented as mean ± SD and median (interquartile range), and categorical variables as *n* (%). Continuous variables were compared using the Mann–Whitney *U*‐test, and categorical variables were compared using Fisher's exact test.

Outcomes were compared between induction of labor at 40 weeks (i.e. 40 + 0 to 40 + 6 weeks) and expectant management, and between induction at 40–41 weeks (i.e. ≥ 40 + 0 weeks) and spontaneous labor. The cumulative incidence of CD types from 40 + 0 weeks onward was analyzed.

Univariable and multivariable logistic regression analyses were used to assess the associations of study variables with IFC, CD‐CTG and CD‐FP. Adjusted odds ratios (aORs) with 95% CI were calculated for key variables, including maternal age and CPR MoM. Statistical analysis was conducted using GraphPad Prism version 9.5® (GraphPad Software, San Diego, CA, USA) and StatPlus Pro version 8® (AnalystSoft Inc., Alexandria, VA, USA); two‐sided *P* < 0.05 was considered statistically significant.

## RESULTS

A total of 739 singleton pregnancies were included in the analysis (Figure 1). Among these, 102 (13.8%) underwent induction of labor at 40 + 0 to 40 + 6 weeks, while 637 (86.2%) were initially managed expectantly. Overall, 393 (53.2%) women experienced spontaneous onset of labor ≥ 40 + 0 weeks, while 346 (46.8%) required induction, the majority of which (70.5%) occurred at ≥ 41 + 0 weeks.

**Figure 1:**
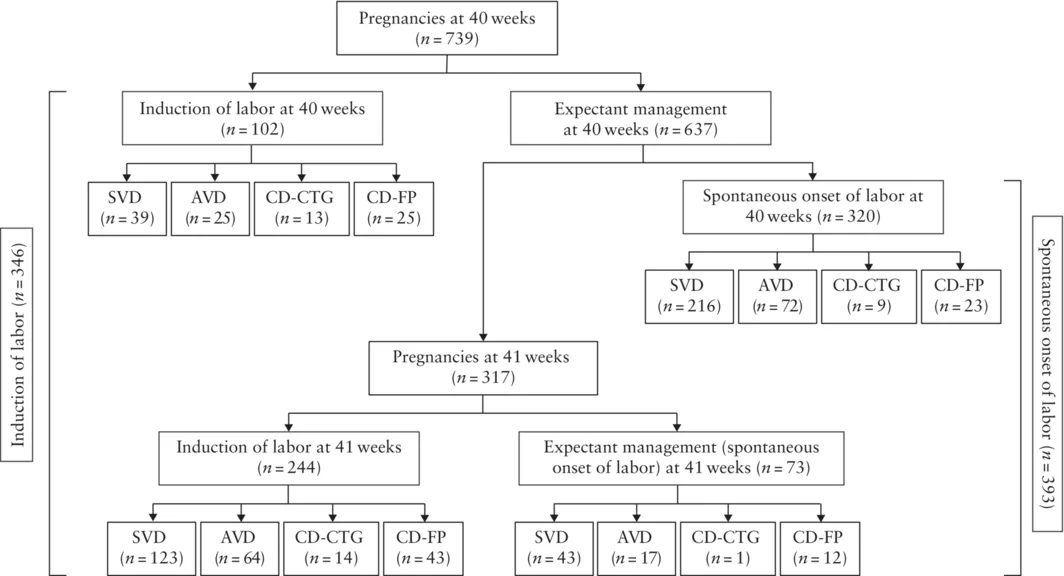
Flowchart showing number and outcome of pregnancies that were induced or progressed to spontaneous onset of labor at 40 and 41 weeks' gestation. AVD, assisted vaginal delivery; CD‐CTG, Cesarean delivery for abnormal cardiotocography; CD‐FP, Cesarean delivery for failure to progress; SVD, spontaneous vaginal delivery.

### Characteristics of study population

Baseline characteristics and pregnancy outcomes of the study population are summarized in Table 1. The mean maternal age was 31.5 ± 5.2 years, with a mean prepregnancy weight of 64 ± 12 kg, height of 163.4 ± 6.4 cm and body mass index of 24.5 ± 4.2 kg/m^2^. Over half (59.1%) of cases were nulliparous. The mean GA at ultrasound examination and delivery were 40.2 ± 0.3 weeks and 40.8 ± 0.3 weeks, respectively. The mean EFW centile was 49.8 ± 29.1, while the mean birth‐weight centile was 41.9 ± 28.2, indicating a tendency toward overestimation of fetal weight. SGA and LGA were observed in 13.4% (99/739) and 5.5% (41/739) of neonates, respectively. IFC, CD‐CTG and CD‐FP occurred in 6.1% (45/739), 5.0% (37/739) and 13.9% (103/739) of attempted labors, respectively. Neonatal outcome was generally favorable: only 0.7% (5/739) of neonates had a 5‐min Apgar score of < 7, and only 1.9% (14/739) had an UA pH of < 7.10.

**Table 1:** Baseline characteristics and pregnancy outcomes of 739 singleton pregnancies that reached 40 + 0 weeks' gestation included in study population

Variable	Value
Maternal age (years)	31.5 ± 5.2; 32 (28–35)
Maternal prepregnancy weight (kg)	64 ± 12; 63 (56–70)
Maternal height (cm)	163.4 ± 6.4; 163 (160–168)
Maternal BMI (kg/m^2^)	24.5 ± 4.2; 23.4 (20.8–26.0)
Parity	0.53 ± 0.77; 0 (0–1)
Nulliparous	437 (59.1)
Smoker	84 (11.4)
Male fetal sex	374 (50.6)
GA at ultrasound (weeks)	40.2 ± 0.3; 40.1 (40.0–40.4)
GA at delivery (weeks)	40.8 ± 0.3; 40.9 (40.4–41.0)
Days from ultrasound to delivery	3.9 ± 2.1; 4 (2–6)
EFW at ultrasound (g)*	3416 ± 389; 3412 (3174–3656)
EFW centile†	49.8 ± 29.1; 48 (25–75)
MCA‐PI MoM	1.02 ± 0.24; 1.00 (0.85–1.18)
UA‐PI MoM	1.08 ± 0.21; 1.06 (0.93–1.20)
CPR MoM	1.03 ± 0.30; 1.00 (0.81–1.20)
BW (g)	3402 ± 374; 3400 (3140–3650)
BW centile†	41.9 ± 28.2; 38 (17–64)
SGA (BW < 10^th^ centile†)	99 (13.4)
LGA (BW > 90^th^ centile†)	41 (5.5)
5‐min Apgar score < 7	5 (0.7)
UA pH < 7.10	14 (1.9)
Onset of labor	
Induction‡	346 (46.8)
Spontaneous‡	393 (53.2)
Mode of delivery	
Spontaneous vaginal delivery	421 (57.0)
Assisted vaginal delivery	178 (24.1)
Cesarean delivery	140 (18.9)
For abnormal CTG	37 (5.0)
For failure to progress	103 (13.9)
Intrapartum fetal compromise	45 (6.1)

Data are given as mean ± SD; median (interquartile range) or as *n* (%).

* According to Hadlock‐4 formula.

† According to local population reference of Hospital Clinic de Barcelona, Barcelona, Spain^15^.

‡ At any time ≥ 40 weeks' gestation. BMI, body mass index; BW, birth weight; CPR, cerebroplacental ratio; CTG, cardiotocography; EFW, estimated fetal weight; GA, gestational age; LGA, large‐for‐gestational age; MCA, middle cerebral artery; MoM, multiples of the median; PI, pulsatility index; SGA, small‐for‐gestational age; UA, umbilical artery.

#### *Induction* vs *expectant management at 40 weeks*

The characteristics and outcomes of pregnancies that underwent induction of labor, compared with those of women who were managed expectantly, at 40 + 0 to 40 + 6 weeks are shown in Table 2. Fetuses in the induction group were assessed and delivered earlier, had lower birth weights, birth‐weight centile, EFW and EFW centile and demonstrated less favorable cerebral and umbilical Doppler parameters compared with those managed expectantly. Additionally, cases that underwent induction of labor were more likely to experience IFC and to result in CD‐CTG or CD‐FP, leading to a higher overall CD rate (*P* < 0.0001).

**Table 2:** Characteristics of study population (*n* = 739), according to whether women were induced or managed expectantly at 40 + 0 to 40 + 6 weeks' gestation

Variable	Induction at 40 weeks (*n* = 102)	Expectant management at 40 weeks (*n* = 637)	*P*
Maternal age (years)	32.3 ± 5.1; 32.5 (29–36)	31.4 ± 5.2; 32 (28–35)	0.1345
Maternal prepregnancy weight (kg)	62 ± 9; 63 (55–70)	64 ± 13; 62 (56–70)	0.5200
Maternal height (cm)	163.7 ± 5.7; 163 (160–168)	163.3 ± 6.5; 163 (160–168)	0.7253
Maternal BMI (kg/m^2^)	23.25 ± 3.7; 22.9 (20.7–25.3)	24.2 ± 4.4; 23.45 (21.0–27.0)	0.2484
Parity	0.45 ± 0.75; 0 (0–1)	0.54 ± 0.78; 0 (0–1)	0.2160
Nulliparous	66 (64.7)	371 (58.2)	0.2340
Smoker	8 (7.8)	76 (11.9)	0.3920
Male fetal sex	55 (53.9)	319 (50.1)	0.5225
GA at ultrasound (weeks)	40.1 ± 0.2; 40.0 (40.0–40.3)	40.2 ± 0.3; 40.1 (40.0–40.4)	0.0008
GA at delivery (weeks)	40.5 ± 0.3; 40.6 (40.3–40.7)	40.8 ± 0.3; 40.9 (40.6–41.0)	< 0.0001
Days from ultrasound to delivery	2.7 ± 1.7; 2 (1–4)	4.1 ± 2.1; 4 (2–6)	< 0.0001
EFW at ultrasound (g)*	3256 ± 451; 3222 (2908–3602)	3442 ± 372; 3422 (3214–3667)	< 0.0001
EFW centile†	39.7 ± 32.4; 33 (8.5–72)	51.4 ± 28.2; 50 (28–76)	0.0001
MCA‐PI MoM	0.96 ± 0.26; 0.92 (0.79–1.11)	1.03 ± 0.23; 1.01 (0.86–1.19)	0.0020
UA‐PI MoM	1.15 ± 0.24; 1.08 (0.99–1.28)	1.07 ± 0.20; 1.06 (0.92–1.19)	0.0080
CPR MoM	0.93 ± 0.32; 0.90 (0.68–1.09)	1.05 ± 0.29; 1.02 (0.85–1.22)	0.0001
BW (g)	3292 ± 446; 3300 (2946–3553)	3419 ± 359; 3410 (3155–3650)	0.0030
BW centile†	37.5 ± 30.4; 33 (10–60)	42.6 ± 27.8; 39 (19–66)	0.0439
SGA (BW < 10^th^ centile†)	25 (24.5)	74 (11.6)	0.0009
LGA (BW > 90^th^ centile†)	6 (5.9)	35 (5.5)	0.8171
5‐min Apgar score < 7	3 (2.9)	2 (0.3)	0.0207
UA pH < 7.10	3 (2.9)	11 (1.7)	0.4250
Onset of labor			
Induction‡	102 (100)	244 (38.3)	< 0.0001
Spontaneous‡	0 (0)	393 (61.7)	< 0.0001
Mode of delivery			
Spontaneous vaginal delivery	39 (38.2)	382 (60.0)	< 0.0001
Assisted vaginal delivery	25 (24.5)	153 (24.0)	0.9012
Cesarean delivery	38 (37.3)	102 (16.0)	< 0.0001
For abnormal cardiotocography	13 (12.7)	24 (3.8)	0.0006
For failure to progress	25 (24.5)	78 (12.2)	0.0018
Intrapartum fetal compromise	13 (12.7)	32 (5.0)	0.0059

Data are given as mean ± SD; median (interquartile range) or *n* (%).

* According to Hadlock‐4 formula.

† According to local population reference of Hospital Clinic de Barcelona, Barcelona, Spain^15^.

‡ At any time ≥ 40 weeks' gestation. BMI, body mass index; BW, birth weight; CPR, cerebroplacental ratio; EFW, estimated fetal weight; GA, gestational age; LGA, large‐for‐gestational age; MCA, middle cerebral artery; MoM, multiples of the median; PI, pulsatility index; SGA, small‐for‐gestational age; UA, umbilical artery.

#### *Induction* vs *spontaneous onset of labor*

The characteristics and outcomes of women who underwent induction of labor, compared with those of pregnancies that progressed to spontaneous onset of labor at any GA ≥ 40 + 0 weeks, are shown in Table 3. Women in the induction group were assessed and delivered later and also had lower birth weight, birth‐weight centile, EFW and EFW centile compared with those who progressed to spontaneous labor. Moreover, induction was associated with higher rates of IFC, CD‐CTG and CD‐FP, resulting in a higher overall CD rate (*P* < 0.0001).

**Table 3:** Characteristics of study population (*n* = 739), according to whether women were induced or progressed to spontaneous onset of labor at ≥ 40 weeks' gestation

Variable	Induction of labor (*n* = 346)	Spontaneous onset of labor (*n* = 393)	*P*
Maternal age (years)	31.7 ± 5.3; 32 (28–35)	31.4 ± 5.0; 32 (28–35)	0.4526
Maternal prepregnancy weight (kg)	63.5 ± 12; 63 (55–70)	64 ± 12; 62 (57–70)	0.5294
Maternal height (cm)	163.4 ± 6.4; 163 (159–168)	163.4 ± 6.4; 163 (160–168)	0.9309
Maternal BMI (kg/m^2^)	23.8 ± 4.2; 22.9 (20.7–26.3)	24.1 ± 4.2; 23.5 (21.2–26.4)	0.4070
Parity	0.44 ± 0.76; 0 (0–1)	0.60 ± 0.78; 0 (0–1)	0.0006
Nulliparous	228 (65.9)	209 (53.2)	0.0006
Smoker	34 (9.8)	50 (12.7)	0.2458
Male fetal sex	169 (48.8)	205 (52.2)	0.3771
GA at ultrasound (week)	40.3 ± 0.3; 40.1 (40.0–40.6)	40.1 ± 0.2; 40.0 (40.0–40.3)	< 0.0001
GA at delivery (week)	40.9 ± 0.3; 41.0 (40.9–41.1)	40.6 ± 0.3; 40.7 (40.4–40.9)	< 0.0001
Days from ultrasound to delivery	4.4 ± 2.1; 5 (3–7)	3.4 ± 2.0; 3 (2–5)	< 0.0001
EFW at ultrasound (g)*	3387 ± 422; 3359 (3098–3643)	3441 ± 355; 3426 (3219–3665)	0.0387
EFW centile†	46.6 ± 30.3; 45 (18–72)	52.6 ± 27.8; 51 (31–77)	0.0054
MCA‐PI MoM	1.04 ± 0.25; 1.02 (0.86–1.19)	1.01 ± 0.22; 1.00 (0.84–1.16)	0.2593
UA‐PI MoM	1.10 ± 0.23; 1.06 (0.95–1.23)	1.06 ± 0.19; 1.06 (0.91–1.19)	0.1449
CPR MoM	1.03 ± 0.31; 1.02 (0.81–1.21)	1.04 ± 0.28; 0.99 (0.83–1.20)	0.8189
BW (g)	3374 ± 397; 3350 (3098–3621)	3426 ± 351; 3430 (3160–3655)	0.0325
BW centile†	38.1 ± 28.5; 32 (13–59)	45.2 ± 27.5; 44 (21–68.5)	0.0003
SGA (BW < 10^th^ centile†)	59 (17.1)	40 (10.2)	0.0068
LGA (BW > 90^th^ centile†)	20 (5.8)	21 (5.3)	0.8725
5‐min Apgar score < 7	5 (1.4)	0 (0)	0.0222
UA pH < 7.10	8 (2.3)	6 (1.5)	0.5904
Mode of delivery			
Spontaneous vaginal delivery	162 (46.8)	259 (65.9)	< 0.0001
Assisted vaginal delivery	89 (25.7)	89 (22.6)	0.3438
Cesarean delivery	95 (27.5)	45 (11.5)	< 0.0001
For abnormal cardiotocography	27 (7.8)	10 (2.5)	0.0012
For failure to progress	68 (19.7)	35 (8.9)	< 0.0001
Intrapartum fetal compromise	30 (8.7)	15 (3.8)	0.0082

Data are given as mean ± SD; median (interquartile range) or *n* (%).

* According to Hadlock‐4 formula.

† According to local population reference of Hospital Clinic de Barcelona, Barcelona, Spain^15^. BMI, body mass index; BW, birth weight; CPR, cerebroplacental ratio; EFW, estimated fetal weight; GA, gestational age; LGA, large‐for‐gestational age; MCA, middle cerebral artery; MoM, multiples of the median; PI, pulsatility index; SGA, small‐for‐gestational age; UA, umbilical artery.

### Cumulative incidence of Cesarean delivery

The cumulative incidence of CD at ≥ 40 weeks' gestation is shown in Figure 2. Induction of labor was associated with a markedly higher cumulative incidence of CD, both for CTG abnormalities and for failure to progress, approximately double the cumulative incidence associated with spontaneous onset of labor. A similar analysis was not performed for induction *vs* expectant management at 40 + 0 to 40 + 6 weeks, as these groups differed in GA at delivery.

**Figure 2:**
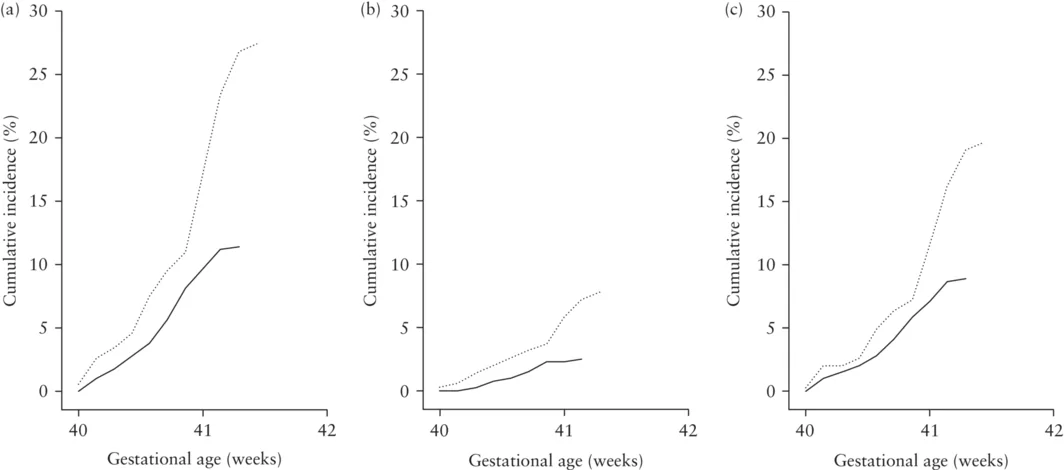
Cumulative incidence of Cesarean delivery (CD) overall (a), CD for abnormal cardiotocography (b) and CD for failure to progress (c) at ≥ 40 weeks' gestation, among pregnancies that were induced (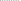) or progressed to spontaneous onset of labor (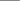).

### Logistic regression analysis

On multivariable logistic regression analysis, both induction of labor and CPR MoM were associated significantly with IFC (aOR, 2.30 (95% CI, 1.20–4.39); *P* = 0.0116 and 0.07 (95% CI, 0.02–0.24); *P* < 0.0001, respectively) and CD‐CTG (aOR, 3.10 (95% CI, 1.46–6.56); *P* = 0.0032 and 0.05 (95% CI, 0.01–0.20); *P* < 0.0001, respectively) (Tables 4 and 5, Figure 3). Meanwhile, parity (aOR, 0.48 (95% CI, 0.32–0.72); *P* = 0.0004), EFW centile (aOR, 1.01 (95% CI, 1.00–1.02); *P* = 0.0018) and induction of labor (aOR, 2.39 (95% CI, 1.54–3.73); *P* = 0.0001) were associated independently with CD‐FP (Table 6, Figure 3).

**Table 4:** Univariable and multivariable logistic regression analyses for prediction of intrapartum fetal compromise ≥ 40 weeks' gestation

	Univariable logistic regression analysis				Multivariable logistic regression analysis*				Final model†			
Variable	Estimate	SE	OR (95% CI)	*P*	Estimate	SE	aOR (95% CI)	*P*	Estimate	SE	aOR (95% CI)	*P*
Maternal age‡	0.0598	0.0312	1.0616 (0.9986–1.1287)	0.0555	—	—	—	—	—	—	—	—
Maternal BMI§	0.0069	0.0471	1.0070 (0.9182–1.1044)	0.8821	—	—	—	—	—	—	—	—
Parity¶	−0.1133	0.2165	0.8929 (0.5841–1.3648)	0.6006	—	—	—	—	—	—	—	—
Male fetal sex	0.3079	0.3112	1.3606 (0.7393–2.5039)	0.3225	—	—	—	—	—	—	—	—
Smoker	0.3848	0.5708	1.4693 (0.4799–4.4977)	0.5003	—	—	—	—	—	—	—	—
Induction of labor	0.8723	0.3253	2.3924 (1.2646–4.5261)	0.0073	0.7888	0.3326	2.2008 (1.1466–4.2242)	0.0177	0.8328	0.3299	2.2999 (1.2045–4.3913)	0.0116
EFW centile¶	−0.0114	0.0055	0.9887 (0.9781–0.9994)	0.0380	−0.0057	0.0056	0.9943 (0.9834–1.0053)	0.3098	—	—	—	—
CPR MoM¶	−2.7885	0.6472	0.0615 (0.0173–0.2187)	< 0.0001	−2.5658	0.6436	0.0768 (0.0218–0.2713)	< 0.0001	−2.6814	0.6364	0.0685 (0.0197–0.2383)	< 0.0001

Multivariable analysis included all parameters that were significant on univariable analysis, while final model selected only those parameters that were significant on multivariable analysis.

* Intercept, −0.4918; Akaike information criterion (AIC), 317.3; area under receiver‐operating‐characteristics curve (AUC), 0.72 (95% CI, 0.65–0.79), *P* < 0.0001; detection rate (DR) at 5% false‐positive rate (FPR), 13% (95% CI, 5–27%); DR at 10% FPR, 36% (95% CI, 22–51%).

† Intercept, −0.6756; AIC, 316.8; AUC, 0.72 (95% CI, 0.65–0.79), *P* < 0.0001; DR at 5% FPR, 20% (95% CI, 10–35%); DR at 10% FPR, 33% (95% CI, 20–49%).

‡ Per 1‐year increase.

§ Per 1‐kg/m^2^ increase.

¶ Per 1‐unit increase. aOR, adjusted odds ratio; BMI, body mass index; CPR, cerebroplacental ratio; EFW, estimated fetal weight; MoM, multiples of the median; OR, odds ratio; SE, standard error.

**Table 5:** Univariable and multivariable logistic regression analyses for prediction of Cesarean delivery for abnormal cardiotocography ≥ 40 weeks' gestation

	Univariable logistic regression analysis				Multivariable logistic regression analysis*				Final model†			
Variable	Estimate	SE	OR (95% CI)	*P*	Estimate	SE	aOR (95% CI)	*P*	Estimate	SE	aOR (95% CI)	*P*
Maternal age‡	0.0729	0.0346	1.0756 (1.0050–1.1511)	0.0353	0.06678	0.0352	1.0691 (0.9976–1.1456)	0.05831	—	—	—	—
Maternal BMI§	0.0468	0.0503	1.0479 (0.9496–1.1564)	0.3519	—	—	—	—	—	—	—	—
Parity¶	−0.4852	0.2973	0.6155 (0.3437–1.1023)	0.1026	—	—	—	—	—	—	—	—
Male fetal sex	0.2605	0.3403	1.2976 (0.6659–2.5283)	0.4439	—	—	—	—	—	—	—	—
Smoker	0.4120	0.6522	1.5099 (0.4205–5.4217)	0.5276	—	—	—	—	—	—	—	—
Induction of labor	1.1761	0.3779	3.2417 (1.5457–6.7986)	0.0019	1.03815	0.3872	2.8239 (1.3221–6.0322)	0.00734	1.1302	0.3829	3.0964 (1.4618–6.5589)	0.0032
EFW centile¶	−0.0166	0.0062	0.9835 (0.9716–0.9956)	0.0076	−0.01141	0.0063	0.9886 (0.9764–1.0010)	0.07249	—	—	—	—
CPR MoM¶	−3.1505	0.7264	0.0428 (0.0103–0.1779)	< 0.0001	−2.71137	0.7238	0.0664 (0.0161–0.2745)	0.00018	−2.9917	0.7103	0.0502 (0.0125–0.2020)	< 0.0001

Multivariable analysis included all parameters that were significant on univariable analysis, while final model selected only those parameters that were significant on multivariable analysis.

* Intercept, −2.69336; Akaike information criterion (AIC), 265.2; area under receiver‐operating‐characteristics curve (AUC), 0.76 (95% CI, 0.70–0.84), *P* < 0.0001; detection rate (DR) at 5% false‐positive rate (FPR), 30% (95% CI, 16–47%); DR at 10% FPR, 38% (95% CI, 23–55%).

† Intercept, −0.8309; AIC, 267.8; AUC, 0.76 (95% CI, 0.68–0.83), *P* < 0.0001; DR at 5% FPR, 24% (95% CI, 12–41%); DR at 10% FPR, 40% (95% CI, 25–58%).

‡ Per 1‐year increase.

§ Per 1‐kg/m^2^ increase.

¶ Per 1‐unit increase. aOR, adjusted odds ratio; BMI, body mass index; CPR, cerebroplacental ratio; EFW, estimated fetal weight; MoM, multiples of the median; OR, odds ratio; SE, standard error.

**Figure 3:**
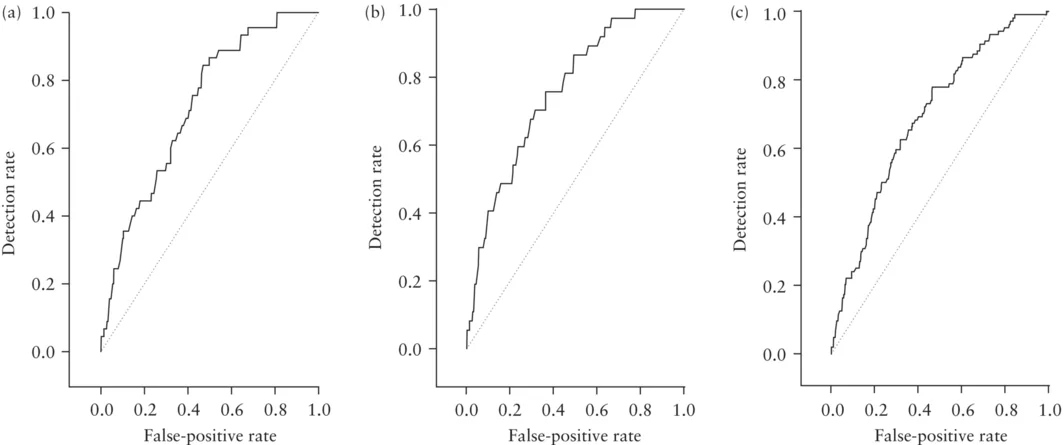
Receiver‐operating‐characteristics (ROC) curves for final models predicting: (a) intrapartum fetal compromise ≥ 40 weeks (area under ROC curve (AUC), 0.72 (95% CI, 0.65–0.79)); (b) Cesarean delivery for abnormal cardiotocography ≥ 40 weeks (AUC, 0.76 (95% CI, 0.70–0.84)); and (c) Cesarean delivery for failure to progress ≥ 40 weeks (AUC, 0.69 (95% CI, 0.64–0.74)).

**Table 6:** Univariable and multivariable logistic regression analyses for prediction of Cesarean delivery for failure to progress ≥ 40 weeks' gestation

	Univariable logistic regression analysis				Multivariable logistic regression analysis*			
Variable	Estimate	SE	OR (95% CI)	*P*	Estimate	SE	aOR (95% CI)	*P*
Maternal age†	−0.0031	0.0205	0.9969 (0.9577–1.0377)	0.8785	—	—	—	—
Maternal BMI‡	0.0773	0.0405	1.0803 (0.9978–1.1696)	0.0566	—	—	—	—
Parity§	−0.7434	0.2035	0.4755 (0.3191–0.7085)	0.0003	−0.7362	0.20660	0.4789 (0.3194–0.7180)	0.0004
Male fetal sex	0.1960	0.2124	1.2166 (0.8023–1.8448)	0.3560	—	—	—	—
Smoker	0.1590	0.5106	1.1724 (0.4309–3.1891)	0.7554	—	—	—	—
Induction of labor	0.8861	0.2211	2.4257 (1.5726–3.7413)	< 0.0001	0.8729	0.22654	2.3939 (1.5356–3.7319)	0.0001
EFW centile§	0.0086	0.0037	1.0086 (1.0014–1.0159)	0.0195	0.0118	0.00379	1.0119 (1.0044–1.0194)	0.0018
CPR MoM§	−0.0142	0.3528	0.9859 (0.4938–1.9685)	0.9679	—	—	—	—

Multivariable analysis (final model) included all parameters that were significant on univariable analysis.

* Intercept, −2.6039; Akaike information criterion, 566.1; area under receiver‐operating‐characteristics curve, 0.69 (95% CI, 0.64–0.74), *P* < 0.0001; detection rate (DR) at 5% false‐positive rate (FPR), 14% (95% CI, 8–23%); DR at 10% FPR, 24% (95% CI, 16–33%).

† Per 1‐year increase.

‡ Per 1‐kg/m^2^ increase.

§ Per 1‐unit increase. aOR, adjusted odds ratio; BMI, body mass index; CPR, cerebroplacental ratio; EFW, estimated fetal weight; MoM, multiples of the median; OR, odds ratio; SE, standard error.

Maternal age was not associated independently with CD‐FP (*P* = 0.8785). Although borderline significant associations were observed on univariable analysis with IFC (*P* = 0.0555) and CD‐CTG (*P* = 0.0353), these associations were no longer significant after adjustment for CPR MoM (*P* = 0.1219 and *P* = 0.0819, respectively) (Table 7). In contrast, CPR MoM emerged as the strongest independent predictor of both IFC and CD‐CTG (both *P* < 0.0001).

**Table 7:** Association of maternal age with intrapartum fetal compromise (IFC) and Cesarean delivery (CD) ≥ 40 weeks' gestation, before and after adjustment for cerebroplacental ratio (CPR) multiples of the median (MoM)

	Univariable logistic regression analysis				Multivariable logistic regression analysis			
Outcome/ variable	Estimate	SE	OR (95% CI)	*P*	Estimate	SE	aOR (95% CI)	*P*
IFC								
Maternal age*	0.0598	0.0312	1.0616 (0.9986–1.1287)	0.0555	0.0488	0.0316	1.0501 (0.9870–1.1171)	0.1219
CPR MoM†	−2.7885	0.6472	0.0615 (0.0173–0.2187)	< 0.0001	−2.7203	0.6505	0.0659 (0.0184–0.2357)	< 0.0001
CD‐CTG								
Maternal age*	0.0729	0.0346	1.0756 (1.0050–1.1511)	0.0353	0.06111	0.03513	1.0630 (0.9923–1.1388)	0.0819
CPR MoM†	−3.1505	0.7264	0.0428 (0.01031–0.1779)	< 0.0001	−3.07140	0.73176	0.0464 (0.0110–0.1945)	< 0.0001
CD‐FP								
Maternal age*	−0.0031	0.0205	0.9969 (0.9577–1.0377)	0.8785	—	—	—	—
CPR MoM†	−0.0142	0.3528	0.9859 (0.4938–1.9685)	0.9679	—	—	—	—

Multivariable analysis for IFC: intercept, −1.7507; Akaike information criterion (AIC), 321.1; area under receiver‐operating‐characteristics curve (AUC), 0.71 (95% CI, 0.64–0.79), *P* < 0.0001; detection rate (DR) at 5% false‐positive rate (FPR), 16% (95% CI, 7–30%); DR at 10% FPR, 30% (95% CI, 16–44%). Multivariable analysis for CD for abnormal cardiotocography (CTG): intercept, −2.07168; AIC, 274.4; AUC, 0.74 (95% CI, 0.65–0.82), *P* < 0.0001; DR at 5% FPR, 19% (95% CI, 8–35%); DR at 10% FPR, 30% (95% CI, 16–47%).

* Per 1‐year increase.

† Per 1‐unit increase. aOR, adjusted odds ratio; FP, failure to progress; OR, odds ratio; SE, standard error.

## DISCUSSION

In this cohort of singleton pregnancies reaching 40 weeks' gestation, induction of labor was associated independently with IFC, CD‐CTG and CD‐FP compared with initial expectant management and spontaneous onset of labor. Importantly, abnormal CPR MoM was also associated independently with IFC and CD‐CTG, whereas CD‐FP was associated additionally with parity and EFW centile. Maternal age was not associated independently with CD or IFC after adjustment for CPR MoM, suggesting that fetal hemodynamic status may account for some of the risk traditionally attributed to advanced maternal age.

### Interpretation of findings and comparison with previous studies

Although our findings cannot be extrapolated directly to earlier gestational weeks, they provide an observational perspective on the relationship between induction of labor and CD at and beyond 40 weeks' gestation^1^, ^4^. The consistent association between induction of labor and CD for both indications suggests that pregnancies selected for induction represent a subgroup at increased risk for CD^21^, ^22^, ^23^. However, because induction is frequently undertaken in pregnancies with specific clinical indications, the observational design of the present study did not allow determination of whether this association was attributable to induction itself or reflected underlying baseline risk factors, including confounding by indication. This interpretation is consistent with evidence from randomized controlled trials and meta‐analyses, which have generally shown no increase in CD following induction at term^2^, ^4^, ^5^, ^6^.

The strong and independent association between abnormal CPR MoM and both IFC and CD‐CTG supports the concept that impaired late‐gestation fetal adaptation to placental insufficiency may play an important role in intrapartum outcome, particularly in the presence of unfavorable cervical conditions^24^, ^25^, ^26^. A systematic review reported that low CPR is associated with a higher risk of emergency CD for abnormal CTG, reinforcing the clinical relevance of CPR in assessing fetal compromise prior to the onset of labor^27^.

Two studies that investigated the predictive value of CPR measured shortly before induction reported that low CPR is associated with increased risks of CD for presumed fetal distress and adverse neonatal outcome^28^, ^29^. These findings support the integration of fetal Doppler assessment into clinical decision‐making in pregnancies reaching the estimated due date, beyond traditional obstetric variables.

Our findings extend this evidence by suggesting that CPR may account partially for the apparent association between advanced maternal age and adverse intrapartum outcome, as the initial associations of maternal age with IFC and CD‐CTG were no longer significant after adjustment for CPR MoM. This aligns with previous data suggesting that the increased risk observed in older mothers may be mediated by placental function rather than maternal age itself^30^. Furthermore, the differential predictors identified for CD‐CTG and CD‐FP reinforce the importance of analyzing CD according to specific indications. Consistent with previous research, while IFC and CD‐CTG appear to be driven primarily by placental and fetal circulatory factors, such as CPR MoM^11^, ^25^, ^26^, ^27^, ^28^, ^29^, ^31^, CD‐FP is related more closely to maternal and fetal mechanical factors, including parity, cervical condition and fetal size^32^, ^33^, ^34^. The independent associations between induction of labor and both CD‐CTG and CD‐FP suggest that the relationship between induction and CD may involve different underlying pathways depending on the indication for operative delivery.

### Confounding by indication

The observational design of this study mandates careful interpretation of the association between induction of labor and CD. Because induction was not assigned randomly, residual confounding by indication cannot be excluded completely. Women undergoing induction differed from those managed expectantly in several baseline characteristics, and some of these differences may have contributed to the decision to induce labor.

However, the observed baseline differences do not appear sufficient to explain fully the higher CD rate observed among induced pregnancies. In particular, fetuses in the induction group had lower EFW centile and birth‐weight centile, characteristics that are associated generally with a lower likelihood of CD‐FP. Therefore, some of the baseline differences would theoretically be expected to decrease, rather than increase, the risk of CD. Another argument is that less favorable Doppler findings in the induction group, compared with those initially managed expectantly, may account partly for the higher incidence of CD‐CTG. However, the association between induction of labor and CD was evident not only for CD‐CTG but also for CD‐FP. Because fetal Doppler abnormalities are unlikely to directly explain the latter outcome, this finding suggests that the relationship between induction and CD cannot be attributed entirely to differences in the characteristics of the induced and expectantly managed populations. Consequently, although we acknowledge that residual confounding may have contributed to the observed results, the available data suggest that baseline differences alone are unlikely to account fully for the higher CD rate observed among induced pregnancies. Nevertheless, these findings should be interpreted within the context of the observational design of the study.

### Strengths and limitations

Strengths of this study include its relatively large sample size, detailed characterization of maternal, fetal and intrapartum variables and inclusion of Doppler parameters in multivariable models. Moreover, analyzing CD by indication provides additional clinical relevance. To our knowledge, this is the first study to evaluate the relative contribution of induction of labor and maternal age while considering the role of CPR MoM.

However, several limitations should be acknowledged. The analysis focused on short‐term neonatal outcome, precluding evaluation of long‐term implications. In addition, information regarding cervical status at induction, including Bishop score, was not available consistently and therefore could not be incorporated into the analyses. As cervical favorability is an important determinant of the success of induction and CD risk, some degree of residual confounding related to cervical status cannot be excluded.

### Conclusions

Among pregnancies reaching 40 weeks' gestation, induction of labor was associated with a higher rate of CD within this observational cohort. Abnormal CPR MoM was associated independently with IFC and CD‐CTG and appeared to attenuate the association between maternal age and adverse intrapartum outcome. These findings support further evaluation of individualized management strategies in these pregnancies, incorporating fetal Doppler assessment, rather than relying exclusively on GA or maternal age in clinical decision‐making.

## Data Availability

The data that support the findings of this study are available from the corresponding author upon reasonable request.
